# Synbiotic Effects of *Saccharomyces* *cerevisiae*, Mannan Oligosaccharides, and β-Glucan on Innate Immunity, Antioxidant Status, and Disease Resistance of Nile Tilapia, *Oreochromis niloticus*

**DOI:** 10.3390/antibiotics10050567

**Published:** 2021-05-12

**Authors:** Gamal El-Nobi, Mohammed Hassanin, Alshimaa A. Khalil, Alaa Y. Mohammed, Shimaa A. Amer, Metwally M. Montaser, Mohamed E. El-sharnouby

**Affiliations:** 1 Department of fish diseases and management, Faculty of Veterinary Medicine, Zagazig University, Zagazig 44511, Egypt; geabdelrehem@vet.zu.edu.eg (G.E.-N.); mahassanin@vet.zu.edu.eg (M.H.); alshimaakhalil@zu.edu.eg (A.A.K.); alaayahyamohamed39@gmail.com (A.Y.M.); 2 Department of Nutrition and Clinical Nutrition, Faculty of Veterinary Medicine, Zagazig University, Zagazig 44511, Egypt; 3 Science and Technology Department, Ranyah University College, Taif University, P.O. Box 11099, Taif 21944, Saudi Arabia; m.montaser@tu.edu.sa; 4 Department of Biotechnology, College of Science, Taif University, P.O. Box 11099, Taif 21944, Saudi Arabia; m.sharnouby@tu.edu.sa

**Keywords:** *Oreochromis niloticus*, synbiotic, norfloxacin, innate immunity, antioxidant activity, *Pseudomonas aeruginosa*

## Abstract

Synbiotic (SYN) additives were assessed as an antibiotic alternative on the effects on the nonspecific immune response and disease resistance of *O. niloticus* to *P. aeruginosa*. Healthy fish (n = 120, average initial weight 18 ± 2 g) were allotted randomly into four experimental groups (3 replicates for each); 1) a control group with no additives (CON), 2) basal diet complemented with 0.1 g kg^–1^ diets of norfloxacin, NFLX, 3) basal diet fortified with 1 mL kg^–1^ diet of SYN, and 4) basal diet complemented with a mixture of NFLX and SYN, which was carried out for eight weeks. Results showed a significant increase (*p* < 0.01) in the serum immune parameters (total protein, globulin and albumin, nitric oxide (NO), and lysozyme activity) in the SYN group and the NFLX+SYN group compared with the CON and NFLX groups. The serum glucose, cholesterol, and triglycerides were higher in NFLX and NFLX+SYN groups than the CON and SYN groups. The catalase (CAT), superoxide dismutase, glutathione peroxidase (GPX) activities were significantly augmented in the NFLX+SYN group, followed by the SYN group compared with CON and NFLX groups. The cumulative mortality rate (CMR) of *O. niloticus* following the *P. aeruginosa* challenge was decreased in the SYN group compared to other groups. The results emphasize that synbiotic could be used as a norfloxacin alternative to enhance the related immunological parameters, including antioxidant activity and disease resistance against *P. aeruginosa* infection of *O. niloticus*.

## 1. Introduction

Fish diseases, especially those caused by bacterial pathogens, are important problems in the fish farm industry [1]. *Pseudomonas* infection has been incriminated as one of the common bacterial infections of fishes and appears as an opportunistic and stress-related disease of freshwater fish cultured under intensive environments [2]. Motile *Pseudomonas* species are the most prevalent fish diseases in Egypt [3], causing septicemia, especially in Nile tilapia [2]. They are highly adaptable opportunistic bacteria that survive in different environments [4] and are responsible for the ulcerative syndrome of bacterial hemorrhagic septicemia, tail and fin rot, and ascites [2].

Antibiotics are commonly used to treat fish diseases and improve their growth performance to prevent and control fish diseases [5]. Large quantities of antibiotics are also used in aquaculture for prevention [6]. Fluoroquinolones control bacterial infections that cause urinary, pulmonary, and digestive tract diseases in farmed fish [7]. Norfloxacin (NFLX), a cheap third-generation fluoroquinolone with broad-spectrum action against aerobic Gram-negative, has been used for fish disease control for several years. However, it has a few side effects in fish [8]. NFLX was the antibiotic of choice after applying an antibiotic sensitivity test against *P. aeruginosa* isolated from infected Nile tilapia. Chemotherapy, including antibiotics, decreases the normal flora of fish and biological filters and must be used only as an emergency measure. It can also reduce or prevent the incidence of bacterial pathogens and be hazardous for aquatic animals [5]. It could also lead to many problems as their cumulative residues affect human health [9]. Thus, other successful alternative preventive measures in aquaculture could be considered to prevent the entrance of the pathogen, enhance water quality, reduce the different stressors in the fish environment, and provide good nutrition and immunization. Furthermore, the application of feed additives like probiotics, prebiotics, and synbiotics in aquacultures enhances the fish’s health status [5].

There is a global interest in using feed additives as an alternative to chemotherapy to enhance fish growth, digestive enzyme activities, and immunity, and prevent disease occurrence [10,11]. Synbiotics are formed from a combination of non-digestible food ingredients (prebiotic) and living organisms (probiotics) that increase the growth and improve the health status among aquaculture species [12,13,14,15,16]. Synbiotics provide more beneficial effects in comparison to the addition of only probiotics or prebiotics [9] for fish and aquatic animals as they promote the establishment of live microbes in the gut and stimulate the metabolism of several beneficial bacteria, which improves the host health [9,17]. 

Nile tilapia (*Oreochromis niloticus*) is one of the most important Tilapia species widely cultured in the tropical, subtropical, and temperate regions [18]. The most important goal in the aquaculture industry is enhancing fish growth, health, and immunity during intensive culture. Fish intensification leads to aquatic disease transmission and fish mortality because microbial diseases are predicted to increase [19]. Accordingly, the search for alternatives to control potential infection of *P. aeruginosa* could promote the health of various fish species, promoting their growth and contributing to food safety. This study was designed to assess the potential effect of using synbiotic as a norfloxacin alternative on serum biochemical parameters, antioxidant activity, immune response, and disease resistance of *O. niloticus* against *P. aeruginosa*.

## 2. Materials and Methods

### 2.1. Feed Additives Used

The commercial product Curazole-M as a synbiotic (Rupa Vet Pharma, Cairo, Egypt) was composed of *Saccharomyces cerevisiae* (1.0 × 10^11^ CFU L^–1^), Echinacea (115 g L^–1^), mannan oligosaccharides (35 g L^–1^), β-glucan (25 g L^–1^), Vitamin E (100 g L^–1^), DL-methionine (30 g L^–1^), L-lysine (10 g L^–1^), choline chloride (100 g L^–1^), minerals (30 g L^–1^) and propylene glycol (75 g L^–1^). The antibiotic NORACIN (norfloxacin 400 mg) was procured from El-Amirya Pharma, Cairo, Egypt. 

### 2.2. Fish and Cultural Conditions

The experiment was carried out according to the guidelines of the care and use of animals for scientific purposes research committee, and ethical approval was obtained from Zagazig University (ZU-IACUC/3/F/170/2019).

Healthy *O. niloticus* fingerlings (n = 120) with an initial average weight of 18 ± 2 g were obtained from the Abassa fish farm in the Sharkia governorate, Egypt. The fish were conditioned for 15 days and fed a basic diet (Table 1) until the experiment began. Before starting the experiment, according to the CCAC, a fish health check was performed to establish that the fish were free of pathogens and disease markers [20]. Fish were assigned randomly to 12 glass tanks (96 L) with ten fish per tank filled with chlorine-free tap water. The aquaria were aerated by a central air compressor. The dissolved oxygen was 6.55 mg/L, and the water temperature and pH were 25 ± 0.5 °C and 7.8 ± 0.4, respectively; all were measured daily. Water was exchanged every other day. Ammonia-N, nitrite-N, and water hardness were measured weekly according to APHA [21]. The light period was controlled automatically as 12 light hours: 12 h darkness in the laboratory.

### 2.3. Diets and Experimental Design

The fish were allotted randomly into four experimental groups (3 replicates per group) (10 fish per replicate): (1) a non-additive control group (CON), (2) basal diet complemented with 0.1 g kg^–1^ diet of NFLX, (3) fortified basal diet with 1 mL kg^–1^ diet of SYN, and (4) basal diet complemented with a mixture of NFLX and SYN. Fish were fed twice by hand until satiety at 9 am and 2 pm for eight weeks. The diet was prepared and formulated according to standard requirements for Nile tilapia [22] (Table 1). The ingredients for the meals were mixed, pelletized, dried for 24 h at room temperature, and stored in the refrigerator until use.

### 2.4. Blood Sampling

At the termination of the experiment (8 weeks of feeding), nine fish/group were collected and anesthetized using 100 mg L^–1^ benzocaine solution (Al-Nasr pharmaceutical Chemicals Co., Oubour, Qalyubia, Egypt). Blood samples (3 samples per group, 1 mL per fish) were collected from the caudal blood vessels without anticoagulant for serum separation using sterile syringes (22 Gauge-3 CC-1). The blood was coagulated for 15–20 min at 4 °C in the refrigerator before being centrifuged for 15 min at 1107× *g.* The separated serum was stored at –20 °C until further analysis for biochemical and immunological parameters.

### 2.5. Nonspecific Immune Parameters

The lysozyme activity was determined colorimetrically using the freeze-dried particles of *Micrococcus lysodeikticus*, labeled with Remazol brilliant blue R (blue ML) that acts as a substrate as identified originally by Ellis [23]. The release of soluble blue products was determined spectrophotometrically at 600 nm after the labeled substrate was treated with lysozyme. On incubation with hen egg lysozyme at 40 °C, the blue color was effectively released at pH 7 and ionic strength of 0.2. The assay method produced a linear dose-response curve with a minimum of 0.1 µg serum lysozyme (1 g/mL, 100 L) in the sample.

The concentration of nitric oxide (NO) was measured calorimetrically according to Schmidt et al. [24]. The colorimetric stain (azo dye) was prepared and used for 96-well plates, with an absorbance of 540 nm (±20 nm). The total assay time was 15 min, and the serum sample size was 50 µL.

### 2.6. Serum Biochemical Parameters

The serum total protein and albumin were measured following the methods of Biuret [25] and Reinhold [26], respectively, using a biochemistry kit by spectrophotometry (Systronics, UV–Visible spectrophotometer – 118, IndiaMART, Uttar Pradesh, India) at 540 nm. The serum globulin was calculated by subtracting serum albumin from total serum protein, as mentioned by Coles [27]. A cell-based (quantitative) assay by Trinder [28] was used for colorimetric determination of serum glucose using Abcam diagnostic kits (ab136955), Giza, Egypt. The serum cholesterol levels were determined calorimetrically using Abcam diagnostic kits (ab65390), Giza, Egypt, following Davidson and Nelson [29].

### 2.7. Antioxidant Activity

The catalase activity (CAT) was determined calorimetrically using Abcam diagnostic kits (ab83464) (Giza, Egypt), according to Aebi’s method [30]. The superoxide dismutase activity (SOD) was measured calorimetrically using Abcam diagnostic kits (ab65354, Giza, Egypt), according to Nishikimi et al. [31]. The content of glutathione peroxidase (GPX) was determined calorimetrically using Abcam diagnostic kits (ab102530, Giza, Egypt) according to Pascual’s method [32].

### 2.8. Challenge Test 

*P. aeruginosa* was previously isolated from naturally infected fishes at the Department of Fish Diseases and Management, Faculty of Veterinary Medicine, Zagazig University, and confirmed to be pathogenic for Nile tilapia. *P. aeruginosa* was identified by conventional biochemical tests and the VITEK 2-C15 automated system for bacterial identification (BioMérieux, Marcy-l’Étoile, France) according to the manufacturer’s instructions. At the end of the experiment, all groups were injected intraperitoneally with the pathogenic strain of *P. aeruginosa* at a dose of 0.1 mL cell suspension containing 3 × 10^7^ cells mL^–1^ measured using McFarland standard tubes. Fish clinical signs and mortalities were recorded for 15 days.

### 2.9. Statistical Analysis

The normality of distribution and homogeneity of variances between different treatments were tested using the Kolmogorov–Smirnov test and Bartlett’s test, respectively, and the assumption was achieved (*p* > 0.05). The data were statistically analyzed by ONE-WAY Analysis of Variance (ANOVA) using SPSS version 24. Duncan’s Multiple Range Test was utilized to assess statistical differences between the groups at a significance level of 0.05 [33]. The data were expressed as mean ± standard error (SE).

## 3. Results

### 3.1. Nonspecific Immune Response

Table 2 demonstrates the effect of NFLX and/or SYN supplementation on the proteinogram of Nile tilapia fingerlings. The serum total protein level reduced in NFLX and NFLX + SYN groups compared with the CON group (*p* < 0.05). The serum globulin level significantly reduced in the NFLX group compared with other groups (*p* < 0.05). The serum level of albumin was insignificant in all experimental groups (*p* > 0.05).

The serum nitric oxide level was enhanced in the SYN group, followed by the NFLX+SYN group, CON, and NFLX groups (*p* < 0.05). The serum lysozyme activity was enhanced in the SYN group compared with other groups (*p* < 0.05) (Table 3).

### 3.2. Serum Biochemical Parameters

The effect of NFLX and SYN supplementation on blood biochemical parameters is shown in Table 4. The serum glucose level increased in the NFLX group compared with other groups. The total cholesterol and triglyceride levels in the serum increased in NFLX and NFLX+SYN groups (*p* < 0.05), meanwhile their values were insignificant in the SYN group compared to the CON group.

### 3.3. Antioxidant Status of Nile tilapia

Significant increases in the serum SOD, CAT, and GSH values were detected in NFLX+SYN and SYN compared to CON and NFLX groups (*p* < 0.01) (Table 5).

### 3.4. Challenge with P. aeruginosa

**Figure 1:**
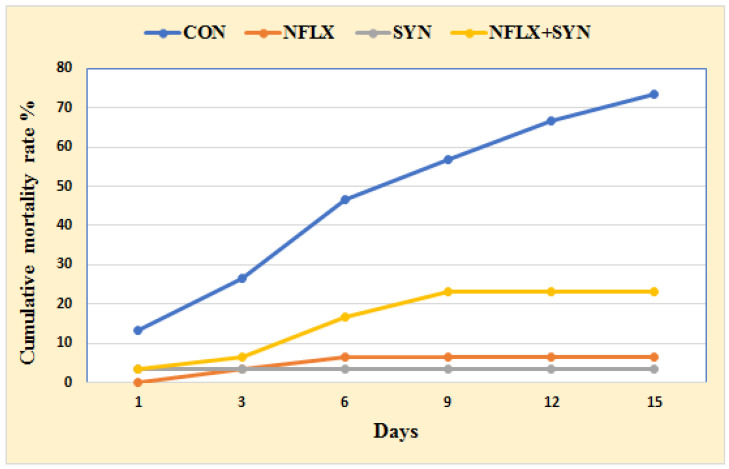
The cumulative mortality rate (%) of Nile tilapia fed with an antibiotic (norfloxacin NFLX) and synbiotic (SYN) after eight weeks and experimentally challenged with *P. aeruginosa*.

Figure 1 shows the impact of dietary additions of NFLX and SYN on Nile tilapia resistance to *P. aeruginosa* challenge in terms of cumulative mortality rate (CMR). The CMR percentage was higher in the SYN group, where the fish were fed an SYN-supplemented diet (3.33%), followed by the NFLX group fed with NFLX-supplemented diet (6.66%), and then the NFLX+SYN group fed with NFLX and SYN in the diet (23.33%). The CMR of the control group was 73.33%.

## 4. Discussion

The combination of a probiotic and prebiotic is known as a synbiotic, which facilitates the colonization and survival of live microbes (probiotics) in the gut, enhancing the health status of the fish [34]. Our study illustrated the potential improvement in the immunological, biochemical, and antioxidant activities of Nile tilapia by synbiotic addition. In our study, the impact of NFLX and SYN dietary addition on the nonspecific immune response of Nile tilapia was studied. The addition of NFLX decreased the serum total protein and globulin, suggesting that NFLX acts as a stress factor on fish and leads to exhaustion of the liver and kidney during metabolism and excretion. Kori-Siakpere et al. [35] proved that the significant decrease in the serum protein due to antibiotic dietary addition indicates tissue damage caused by its toxicity, stress, and hepatic impairment, resulting in impairment of cell membranes, which permits protein to leak out.

There was an increase in the serum total protein and globulin in the SYN-supplemented diet. This may be due to the presence of protease-enzyme released from aged yeast cells (*S. cerevisiae*), which improves the digestibility of protein and inhibits intestinal bacteria toxins. In contrast, the cytotoxic effect of bacterial toxins causes severe hepatic and intestinal tissue damage in the control group [36]. According to Opiyo et al. [37], a high serum protein and albumin level correlate with a potent nonspecific immune response, ensuring the positive effect of a synbiotic on Nile tilapia. The current results elucidated that serum nitric oxide increased in the SYN group compared to the NFLX+SYN group. The SYN group was higher in serum lysozyme level than other groups. The hazardous consequence of using antibiotics, particularly NFLX, as a feed additive was detected on the nonspecific immune response, which could be attributed to liver dysfunction. Other studies showed different results and proved that antibiotics suppress fish immunity [38,39].

Many studies recently proved that synbiotics are essential as immune enhancera and, consequently, disease control, especially in Nile tilapia [40,41]. Our data emphasized the vital role of SYN in promoting the nonspecific immune response, especially for NO and lysozyme serum levels. This may be due to the importance of probiotics, whole yeast cell (*S. cerevisiae*), and yeast cell wall-derived carbohydrates increasing the phagocytic activity, which has a significant role in bacterial defenses and accounts for early activation of the inflammatory response before antibody production [42]. Synbiotic (SYN) also contains mannan-oligosaccharide, which could attach to some Gram-negative bacteria, thereby preventing it from infection, which subsequently increases fish immunity [43]. β-glucan content of SYN can increase lysozyme and nitric oxide production, promoting the immune system of fish, as stated by Adloo et al. [44] and Dawood et al. [45].

*Echinacea purpurea* (EP) is a globally popular herbal medicine known as an immunostimulant in humans, animals, and fish [46]. In fish, dietary supplementation with EP has been reported to enhance the immune response and resistance against bacterial infection [47,48,49] and cause a significant increase in serum lysozymes and bactericidal activity (SBA) [48], thus leading to stimulation of the fish immune system and improvement in health status. These results were concordant with Aly et al. [49], who showed the immunostimulatory effects of EP supplemented diets on *O. niloticus*. Furthermore, the diet fortified with EP resulted in the enhancement of serum total protein and globulins, considered as a strong innate-immune response in fishes [48,50]. These results were concordant with Oskoii et al. [47] and Khalil and Elhady [48], who reported that the total serum protein, albumin, and globulin levels were increased in EP-fed fish. The enhancement observed in the immune function of SYN-supplemented groups may be due to the polysaccharides, glycoproteins, caffeic acid derivatives, alkamides, and isobutyl amides present in *Echinacea* responsible for its immunomodulatory effects [48].

The NFLX group showed an increase in serum glucose, total cholesterol, and triglyceride levels. The SYN addition did not affect these levels compared with the CON group. Shalaby et al. [51] demonstrated a significant rise in serum glucose concentration in Nile tilapia administered with different levels of antibiotics compared with control fish, suggesting that fish obtained more energy to withstand stress conditions since the plasma or serum glucose level is an indicator for nonspecific stress. In addition to that, the high levels of total cholesterol and triglycerides in Nile tilapia were reported after taking different doses of antibiotics. This could be attributed to the chronic exposure of *O. niloticus* to NFLX, resulting in genotoxicity, which leads to physiological alterations such as energy metabolism alterations of liver tissue and muscles of fish [52,53], leading to an increase in protein and a decrease in lipid contents in *O. niloticus*.

Similarly, Peres et al. [54] and Gelibolu et al. [55] found no significant increase in glucose levels between groups fed with a diet supplemented with mannan-oligosaccharides and control groups. In addition, Abu-Elala et al. [36] added yeast with mannan-oligosaccharide to the fish diet and Kuhlwein et al. [56] added yeast and β-glucan to the fish diet, which showed no significant increase in serum glucose concentration after eight weeks of feeding. Adloo et al. [44], Pilarski et al. [57], and Sanchez-Martinez et al. [58] found a decrease in serum glucose levels in groups fed a diet complemented with β-glucan, indicating the ability of β-glucan to increase intestinal viscosity resulting in slow glucose absorption from the bloodstream.

Concerning the effect of NFLX and SYN on the antioxidant activity of Nile tilapia, our results showed a significant increase in the serum antioxidant enzyme levels in the NFLX+SYN group, followed by the SYN group compared to the CON and NFLX group. This may be attributed to the β-glucan content of SYN, which may enhance the antioxidant enzyme levels; glutathione peroxidase (GPX), catalase (CAT), and superoxide dismutase (SOD) compared to the control groups [45]. Many studies supported our data, such as Dalmo and Bøgwald [59], Li et al. [60], Lin et al. [61], and Adloo et al. [44]. Furthermore, Guzmán-Villanueva et al. [62] explained that the enhancement of antioxidant activity might be because β-glucan increases the circulating neutrophils in blood and stimulates phagocytic cells related to the reactivation of oxygen species. NFLX can also stimulate the suppression of cytochrome P450 monooxygenase activity in fish, as reported by Vaccaro et al. [63], thereby prompting the activity of some phase II enzymes, such as glutathione S-transferases (GST), which play a vital role in anti-oxidation through combination with glutathione (GSH). GST enzymes and GSH content activity are used to evaluate the efficiency of a drug’s metabolism, as discussed by Li et al. [64]. However, this does not prevent the harmful effect on the liver and digestive system, explaining the decrease in antioxidant enzymes in the NFLX group.

*Pseudomonas* septicemia is considered a critical fish disease, causing high mortality rates and economic losses among freshwater fishes [65]. The challenge test is used as an ultimate assay to assess the fish immune response [66,67,68,69,70,71]. The results displayed an increase in the survivability of fish fed a SYN diet following challenge with *P. aeruginosa,* indicating improvement of the immunity of fish fed a diet complemented with synbiotic and a competitive efficiency against pathogenic bacteria [40,41] as previously proven by the enhancement of several immunological markers. The highest survival rate after *P. aeruginosa* infection was observed in the SYN group due to the positive effect of *S. cerevisiae*, mannan-oligosaccharide, and β-glucan on the immune response, which resulted in increased bacterial resistance as recorded by Abu-Elala et al. [36] and Okey et al. [43] and the effectiveness of the antimicrobial agent against the pathogen.

## 5. Conclusions

The present results showed the beneficial effect of synbiotics (*Saccharomyces cerevisiae*, mannan oligosaccharides, and β-glucan) in enhancing the immune resistance of fish against *P. aeruginosa* infection. Synbiotics can be used as an alternative to antibiotics to improve blood biochemical parameters and antioxidant activity. The results provided data for a synbiotic application as a useful feed additive in the Nile tilapia aquaculture industry.

## Figures and Tables

**Table 1:** The proximate chemical composition of the basal diet (g kg^–1^, on dry matter basis).

Ingredients	g kg^–1^
Fish meal 70.7% CP	180
Yellow corn	220
Corn gluten 67% CP	90
Soybean meal 49% CP	284
Wheat flour	90
Wheat bran	70
Fish oil	60
Vitamins and minerals mixture ^1^	3.0
Methionine	3.0
Chemical analysis (g kg^–1^)	
Crude protein	373.94
Fat	98.85
Crude fiber	38.43
NFE ^2^	423.25
GE (MJ/kg) ^3^	20.73
Lysine	20.76
Methionine	10.63
Ash	65.51

^1^ Composition of vitamins and minerals premix kg^−1^: vitamin D3 8600 IU; vitamin A 580000 IU; vitamin E 720 mg; vitamin C 0.1 mg; vitamin K3 142 mg; vitamin B1 58 mg; vitamin B2 34 mg; vitamin B6 34 mg; vitamin B12 58 mg; pantothenic acid 8 mg; folic acid 86 mg; biotin 50 mg; zinc methionine 3000 mg; iron sulfate 2000 mg; manganese sulfate 65 mg; copper sulfate 3400 mg; cobalt sulfate 572 mg; sodium selenite 25 mg; calcium iodide 25 mg; calcium carbonate as carrier up to till 1 kg. ^2^ Nitrogen free extract was determined by difference = 100-(protein % + fat % + crude fiber % + ash %). ^3^ Gross energy (GE) was calculated according to NRC [22] as 23.6 KJ/g protein, 39.5 KJ/g lipids and 17.0 KJ/g NFE. CP: Crude protein.

**Table 2:** Comparison of the proteinogram of Nile tilapia, *O. niloticus*, fed with diets containing antibiotic (norfloxacin, NFLX) and synbiotic (SYN).

Groups	Total Protein (g/dL)	Albumin (g/dL)	Globulin (g/dL)
CON	5.90 ± 0.23 ^a^	2.48 ± 0.07	3.16 ± 0.04 ^a,b^
NFLX	3.59 ± 0.47 ^c^	2.50 ± 0.17	1.09 ± 0.31 ^c^
SYN	6.09 ± 0.19 ^a^	2.65 ± 0.28	3.54 ± 0.15 ^a^
NFLX+SYN	5.15 ± 0.15 ^b^	2.45 ± 0.15	2.70 ± 0.30 ^b^

^a, b, c^ Means with different superscripts are statistically different at *p* < 0.05 according to Duncan’s multiple range test. The data are expressed as the mean ± standard error (SE).

**Table 3:** Changes in the serum nitric oxide and lysozyme activities of Nile tilapia, *O. niloticus*, fed diets containing antibiotic (norfloxacin, NFLX) and synbiotic (SYN).

Groups	Nitric Oxide (µmol/L)	Lysozyme (µg/mL)
CON	74.00 ± 2.60 ^c^	18.50 ± 2.50 ^b^
NFLX	77.25 ± 3.35 ^c^	19.00 ± 1.00 ^b^
SYN	97.70 ± 2.20 ^a^	24.50 ± 2.50 ^a^
NFLX+SYN	89.65 ± 1.15 ^b^	20.50 ± 1.50 ^b^

^a, b, c^ Means with different superscripts are statistically different at *p* < 0.05 according to Duncan’s multiple range test. The data are expressed as the mean ± standard error (SE).

**Table 4:** Changes in serum glucose, total cholesterol, and triglycerides in *O. niloticus* fed diets supplemented with antibiotic (norfloxacin, NFLX) and synbiotic (SYN).

Groups	Glucose (mg/dL)	Total Cholesterol (mg/dL)	Triglycerides (mg/dL)
CON	89.96 ± 2.35 ^b^	191.00 ± 4.00 ^c^	165.50 ± 5.50 ^c^
NFLX	111.95 ± 7.75 ^a^	248.00 ± 3.00 ^a^	221.00 ± 10.00 ^a^
SYN	89.65 ± 1.15 ^b^	195.50 ± 4.50 ^c^	170.50 ± 5.50 ^c^
NFLX+SYN	97.55 ± 3.25 ^b^	215.50 ± 5.50 ^b^	200.50 ± 3.50 ^b^

^a, b, c^ Means with different superscripts are statistically different at *p* < 0.05 according to Duncan’s multiple range test. The data are expressed as the mean ± standard error (SE).

**Table 5:** Changes in serum sodium dismutase (SOD), catalase (CAT), and reduced glutathione (GSH) in *O. niloticus* fed diets supplemented with antibiotic (Norfloxacin NFLX) and synbiotic (SYN).

Groups	SOD (U/mL)	CAT (U/L)	GSH (IU/L)
CON	3.95 ± 0.18 ^c^	260.00 ± 11.00 ^b^	147.50 ± 22.50 ^b^
NFLX	4.42 ± 0.35 ^c^	275.50 ± 12.50 ^b^	157.50 ± 7.50 ^b^
SYN	5.31 ± 0.29 ^b^	295.00 ± 6.00 ^a^	187.00 ± 3.00 ^a^
NFLX+SYN	6.36 ± 0.59 ^a^	301.50 ± 5.50 ^a^	209.00 ± 14.00 ^a^

^a, b, c^ Means with different superscripts are statistically different at *p* < 0.05 according to Duncan’s multiple range test. The data are expressed as the mean ± standard error (SE).
